# Opportunities for antimicrobial stewardship in patients with acute bacterial skin and skin structure infections who are unsuitable for beta-lactam antibiotics: a multicenter prospective observational study

**DOI:** 10.1177/2049936118823655

**Published:** 2019-02-04

**Authors:** Jonathan A.T. Sandoe, Kordo Saeed, Achyut Guleri, Kieran S. Hand, Ryan Dillon, Mike Allen, Amazigom Mayes, Fiona Glen, Armando Gonzalez-Ruiz

**Affiliations:** Leeds Teaching Hospitals NHS Trust and University of Leeds, Leeds, UK; Hampshire Hospitals NHS Foundation Trust and University of Southampton, Southampton, UK; Blackpool Teaching Hospitals NHS Foundation Trust, Blackpool, UK; University Hospital Southampton NHS Foundation Trust, Southampton, UK; Merck Sharp & Dohme UK Ltd. (MSD), Hoddesdon, UK; Merck Sharp & Dohme UK Ltd. (MSD), Hoddesdon, UK; Merck Sharp & Dohme UK Ltd. (MSD), Hoddesdon, UK; pH Associates Ltd, Marlow, UK; Darent Valley Hospital, Dartford and Gravesham NHS Trust, Dartford, UK

**Keywords:** acute bacterial skin and skin-structure infection, antibiotics, antimicrobial stewardship, antimicrobials, complicated forms of skin and soft-tissue infections, long-acting antibiotics, methicillin-resistant *Staphylococcus aureus*, outpatient parenteral therapy

## Abstract

**Purpose:**

The objective of this prospective, observational study was to describe the treatment, severity assessment and healthcare resources required for management of patients with acute bacterial skin and skin structure infections who were unsuitable for beta-lactam antibiotic treatments.

**Methods:**

Patients were enrolled across five secondary care National Health Service hospitals. Eligible patients had a diagnosis of acute bacterial skin and skin structure infection and were considered unsuitable for beta-lactam antibiotics (e.g. confirmed/suspected methicillin-resistant *Staphylococcus aureus*, beta-lactam allergy). Data regarding diagnosis, severity of the infection, antibiotic treatment and patient management were collected.

**Results:**

145 patients with acute bacterial skin and skin structure infection were included; 79% (*n* = 115) patients received greater than two antibiotic regimens; median length of the first antibiotic regimen was 2 days (interquartile range of 1–5); median time to switch from intravenous to oral antibiotics was 4 days (interquartile range of 3–8, *n* = 72/107); 25% (*n* = 10/40) patients with Eron class 1 infection had systemic inflammatory response syndrome, suggesting they were misclassified. A higher proportion of patients with systemic inflammatory response syndrome received treatment in an inpatient setting, and their length of stay was prolonged in comparison with patients without systemic inflammatory response syndrome.

**Conclusion:**

There exists an urgent need for more focused antimicrobial stewardship strategies and tools for standardised clinical assessment of acute bacterial skin and skin structure infection severity in patients who are unsuitable for beta-lactam antibiotics. This will lead to optimised antimicrobial treatment strategies and ensure effective healthcare resource utilisation.

## Introduction

Skin and soft tissue infections (SSTIs) are associated with significant morbidity and mortality and are a common reason for antimicrobial prescriptions globally.^1–3^ Penicillins are the recommended first-line therapy for many SSTI, but they cannot always be prescribed, for various reasons, including: infection with resistant pathogens, like methicillin-resistant *Staphylococcus aureus* (MRSA); and penicillin allergy. There is a range of alternative oral and intravenous (IV) antibiotics, but limited evidence to guide choice. Accurate assessment of disease severity can help in selecting effective oral or outpatient parenteral antibiotic treatments. However, despite various criteria being suggested for the assessment of SSTI severity,^4–6^ there are currently no uniform, validated criteria for assessing patients with SSTI.

The ‘Eron’ classification system, which was devised in 2003 from the recommendations of an expert panel, classifies SSTIs based on the severity of local and systemic signs, and it is one of the most widely known classification systems.^4^ The presence of systemic inflammatory response syndrome (SIRS), based on the patient’s temperature, heart rate, respiratory rate and white blood cell (WBC) count, is widely used and may be of value in SSTI.^5,7^ A recent single-centre study showed an association between the presence of SIRS in patients with SSTI and poorer clinical outcomes.^8^

The UK Chief Medical Officer’s report^9^ and subsequent World Health Organization (WHO)^10^ report on the global prevalence of antimicrobial resistance led to a renewed focus on antimicrobial stewardship (AMS) programmes for the judicious use of antimicrobial agents in the United Kingdom. Because SSTI are so common, optimising therapy for these infections is an important component of AMS programmes. Delivering evidence-based and rational prescribing of antibiotics is a priority across the United Kingdom,^11,12^ with outpatient parenteral therapy (OPAT) services playing a key role in the achievement of these goals.^13^

In 2010, the term acute bacterial skin and skin structure infection (ABSSSI) was introduced to help further delineate types of skin infections. The definition of ABSSSI excludes chronic skin and skin structure infections. Infections considered to be ABSSSI include cellulitis/erysipelas, wound infections, or major cutaneous abscesses and exclude less serious skin infections (e.g. impetigo and minor cutaneous abscess), as well as infections needing more complex treatment regimens (e.g. infections resulting from animal or human bites, necrotising fasciitis, diabetic foot infection, decubitus ulcer infection, myonecrosis and ecthyma gangrenosum).^14^

The primary aim of this study was to describe patient population, current treatment and management trends of ABSSSI in the hospital setting where first-line therapy with beta-lactam antibiotics could not be used. The secondary aim of this study was to assess the potential role of different severity assessment tools in the treatment and management of ABSSSI.

## Methods

### Study design and setting

In this prospective observational study (designed and reported according to STROBE criteria),^15^ patients with a diagnosis of ABSSSI were recruited from five secondary care National Health Service (NHS) centres in the United Kingdom. The study observation period extended from the date of presentation with ABSSSI (ranging from December 2015 to November 2016) until the date of death or 30 days post-discharge from hospital care (ranging from January 2016 to January 2017). All data collection was undertaken by suitably qualified members of the care team at each hospital, with a strict requirement to maintain confidentiality. Data collected from study sites were anonymously coded to preserve confidentiality and transferred to pH associates (an independent research consultancy specialising in real-world evidence research). Data analysis was conducted by an independent data analyst who was not involved in data collection.

### Research ethics

All patients gave informed consent, and the study was approved by the South East Coast – Brighton & Sussex Research Ethics Committee (reference no. 15/LO/1249) and the local research and development (R&D) department of each participating NHS centre.

### Participants and selection criteria

Consenting patients aged 18 years or over with a clinical diagnosis of ABSSSI and receiving at least some of their treatment for ABSSSI in hospital were included in the study if they fulfilled one of the following criteria: (a) considered inappropriate for initiation or continuation of beta-lactam antibiotics OR (b) had confirmed MRSA at the site infection or in blood culture OR (c) were suspected to have MRSA infection (in clinician’s opinion).

The criteria for exclusion from the study were as follows: (a) if patient was diagnosed or managed solely in primary care OR (b) patient was unwilling or unable to give consent OR (c) patients were referred to other hospitals for part of their treatment pathway or where treatment data were not available.

According to the definition of ABSSSI, chronic skin and skin structure infections were excluded, as were less serious skin infections (e.g. impetigo and minor cutaneous abscess) and infections needing more complex treatment regimens (e.g. infections resulting from animal or human bites, necrotising fasciitis, diabetic foot infection, decubitus ulcer infection, myonecrosis and ecthyma gangrenosum).^14^

### Study variables

Data relating to patient characteristics, ABSSSI diagnosis, treatment, severity and management pathway were collected from the medical records for each patient included in the study from the date of first presentation with ABSSSI to 30 days post-discharge from hospital care or death (whichever occurs first). Data included in the final analysis were collected between February 2016 and January 2017.

### Severity classification

The results of an ABSSSI severity assessment according to the Eron classification^4^ were collected for each patient (Supplementary Table 1). The clinical characteristics required for assessment of SIRS, body temperature, heart rate, respiratory rate and WBC count were collected from medical records at the time of ABSSSI diagnosis. The criteria for SIRS categorisation^7^ are described in Supplementary Table 2. Only patients with data available for all four clinical characteristics were included in the final SIRS analysis. Patients, who satisfied two or more of each criterion for SIRS, were categorised as having SIRS. This categorisation was performed retrospectively after data collection was complete. Final results comparing SIRS and Eron categorisation were presented for those patients for whom data for both severity categories was available.

Antibiotic treatment regimens were defined according to the date at which treatment was first commenced for ABSSSI in each patient. A patient’s first treatment regimen was defined as the antibiotic (or combination) which was initially started for ABSSSI, and subsequent changes to antibiotic therapy were recorded as new regimens. The duration of each subsequent antibiotic treatment regimen was calculated from the date of completion of the first regimen.

### Bias

Participating centres could recruit from any one of the specified hospital areas and developed local systems for identifying and approaching consecutive eligible patients, to reduce the risk of bias.

### Clinical outcomes

The clinical outcomes analysed in this study were patient demographics, patients’ length of stay (LOS) in hospital, management settings of patients (inpatients *versus* non-inpatients) and infection status of patients at time of discharge and healthcare resource utilisation. ABSSSI-related LOS was defined as the total number of days spent by a patient with ABSSSI under secondary care including management in both inpatient and non-inpatient settings. Patients who were sent home while still receiving antibiotic treatment were defined as patients with ‘resolving’ infections, and patients who were discharged without further continuing antibiotic treatment were defined as patients with ‘resolved’ infections in the final analysis. Data for patients who were fit for discharge but remained as inpatients due to other reasons are described separately.

### Healthcare resource utilisation

Healthcare resource cost per patient of £467.30 per day was calculated from NHS reference costs (2015–2016) for non-elective inpatients for the treatment of infectious diseases, using weighted average unit cost for finished consultant episodes (FCEs) and weighted average LOS in hospital.^16^ The calculations for maximum potential healthcare cost savings were made for the assumption that all ABSSSI-related inpatient days would be replaced by OPAT (costs for OPAT as described previously),^17^ and the calculations for minimum potential cost savings were made assuming a minimum of one inpatient bed day being replaced by OPAT.

### Sample size

As this observational study did not aim to compare patient cohorts, sample size calculations were based on precision estimates (confidence intervals (CIs)) rather than statistical power for significance. Therefore, based on the central limit theorem, assuming a normal distribution of variables, a sample size of 300 was chosen to ensure reliability and precision in estimating the study variables. While it was acknowledged that the results for each individual hospital would be of limited precision for such outcomes, they would have good precision for any quantitative outcomes (e.g. means), as samples of 40 or more generate precise estimates of standard deviations (SDs) and hence the CIs for the means.

The total number of patients finally recruited to the study was 145, lower than the recommended sample size of 300. This was because the number of eligible patients presenting at each centre during the prospective recruitment period was lower than anticipated. Because the outcomes of the study were descriptive and no specific statistical null hypotheses were being tested, the decision was made to proceed with the analysis using the data available, rather than extend the recruitment period.

### Statistical analysis

All statistical analysis was performed using Microsoft Excel^®^ and STATA^®^ v14.1, using available results with no imputation of missing values. Quantitative variables are presented as mean (SD) or median [interquartile range (IQR)], depending on normal distribution of variables or not. Categorical data are presented as frequency (percentage). Where applicable, quantitative variables were compared by the Wilcoxon rank-sum test while differences between categorical variables were assessed using the Chi-square test, with the threshold for significance set at the 5% level.

## Results

### Patient demographics and clinical characteristics

A total of 635 patients were initially identified from five hospitals and screened for inclusion according to the study selection criteria. A total of 258 patients were deemed eligible to participate in the study and were approached for consent. A total of 145 consenting patients were finally recruited to the study. The study population comprised of 52% (*n* = 76) female patients. The mean (±SD) age of patients enrolled in the study was 64 (±17) years. Six percent (*n* = 9/145) of patients had confirmed MRSA infection while 13% (*n* = 19/145) were suspected to have MRSA infections (in the opinion of the clinical team). The remaining (*n* = 117) patients in the study group were deemed unsuitable for initiation (*n* = 92) or unsuitable for continuation of beta-lactam antibiotics (*n* = 25) according to clinician’s judgement. These patient characteristics are described in detail in Table 1.

**Table 1.** Patient demographics and clinical characteristics.

Number of patients, *n*	145
Female sex, *n* (%)	76 (52)
Mean age (±SD), years	64 (17)
Confirmed MRSA, *n* (%)	9 (6)
Suspected MRSA, *n* (%)	19 (13)
Total Inappropriate for beta-lactam antibiotics, *n* (%)	117 (81)
Unsuitable for initiation of beta-lactam antibiotics, *n* (%)	92 (63)
Unsuitable for continuation of beta-lactam antibiotics, *n* (%)^a^	25 (17)
Cellulitis, *n* (%)	116 (80)
Eron classification data available, *n* (%)	103 (71)
Eron 1	55 (38)
Eron 2	46 (32)
Eron 3	2 (1)
Eron 4	0
No Eron classification, *n* (%)	42 (29)
All four SIRS data components available, *n* (%)	120 (83)
SIRS present, *n* (%)	41 (34)

MRSA, methicillin-resistant *Staphylococcus aureus*; SIRS, systemic inflammatory response syndrome.

a 18 patients who met the inclusion criteria on the grounds of being unsuitable for the continuation of beta-lactam treatment received flucloxacillin as part of their first regimen; 11 of these patients received flucloxacillin in combination with other treatments.

### ABSSSI severity assessment, treatment and management

The distribution of Eron and SIRS severity categories for all patients is shown in Table 1. In total, 59% (*n* = 85/145) of patients had data available for both Eron and SIRS classification criteria. A comparison of severity assessments and analysis of treatment and management of ABSSSI for these 85 patients is shown in Table 2. A higher proportion of patients with SIRS received treatment in an inpatient setting in comparison with patients without SIRS (Chi-square test; *p* = 0.001). The differences in the proportion of patients receiving treatment in an inpatient setting were not statistically significant according to Eron classification (*p* = 0.109). The median (IQR) LOS of patients with Eron 1, 2 and 3 classifications was 5.0 (3.0–14.0), 8.0 (4.0–14.0) and 9.0 (7.5–10.5) days, respectively (Eron 1, *n* = 40; Eron 2, *n* = 43; and Eron 3, *n* = 2 as shown in Table 2). A significant difference in LOS was observed between patients with SIRS in comparison to patients without SIRS [SIRS: 14.0 (5.8–20.5) days; non-SIRS: 6.0 (3.0–12.0) days; *p* = 0.0023].

**Table 2.** Treatment and management of patients with different acute bacterial skin and skin structure infections (ABSSSI) severity classifications.

Eron class^a^	SIRS	*n*	Number of antibiotic regimens	First-regimen IV antibiotics, *n* (%)	First-regimen Oral antibiotics, *n* (%)	Inpatients, *n* (%)	Non-inpatients, *n* (%)	Infection status at time of discharge,^b^ *n* (%)	Length of stay (days), median (IQR)
1	SIRS	10	1: 3 (30%)	9 (90%)	1 (10%)	7 (70%)	3 (30%)	Resolved: 5 (50%) Resolving: 5 (50%) Mortality: 0	10.5 (3.3–14.0)
			2: 2 (20%)						
			⩾3: 5 (50%)						
	Non-SIRS	30	1: 4 (13%)	26 (87%)	4 (13%)	8 (27%)	22 (73%)	Resolved: 10 (33%) Resolving: 20 (67%) Mortality: 0	4.5 (3.0–12.8)
			2: 16 (53%)						
			⩾3: 10 (33%)						
2	SIRS	15	1: 1 (7%)	15 (100%)	0	11 (73%)	4 (27%)	Resolved: 8 (53%) Resolving: 6 (40%) Mortality: 1 (7%)^c^	16.0 (7.5–21.5)
			2: 4 (27%)						
			⩾3: 10 (67%)						
	Non-SIRS	28	1: 5 (18%)	22 (79%)	5 (18%)	12 (43%)	16 (57%)	Resolved: 7(25%) Resolving: 20 (71%) Mortality: 1 (4%)^c^	6.0 (4.0–11.0)
			2: 11 (39%)						
			⩾3: 12 (43%)						
3	SIRS	2	1: 0 (0%)	2 (100%)	0	2 (100%)	0 (0%)	Resolved: 0 Resolving: 2 (100%) Mortality: 0	9.0 (7.5–10.5)
			2: 1 (50%)						
			⩾3: 1 (50%)						
	Non-SIRS	0	N/A	N/A	N/A	N/A	N/A	N/A	N/A

SIRS, systemic inflammatory response syndrome; IQR, interquartile range.

a *n* = 0 for Eron 4.

b Includes patients treated as a non-inpatient, that is, time till deemed no longer necessary to receive ABSSSI treatment and related monitoring.

c Both patients who died received treatment as inpatients.

A total of 79% (*n* = 115/145) patients were treated with multiple antibiotic regimens. The antibiotics used (either alone or in combination with other antibiotics) as part of the first treatment regimen and reasons (based on clinician’s judgement) for changes in antibiotic regimens are shown in Supplementary Tables 3 and 4, respectively. The most commonly used first-regimen antibiotics in our study group were teicoplanin (32%, *n* = 47/145) and clindamycin (32%, *n* = 47/145) (Supplementary Table 5).

In total, 27% of patients (39/145) received at least one beta-lactam antibiotic as part of their first regimen (14 of these patients had confirmed or suspected MRSA) and received a non-beta-lactam antibiotic as part of a later regimen. Of the patients who received a beta-lactam antibiotic as part of their first regimen, 26 patients received flucloxacillin (11 patients received it as a single agent while 15 patients received flucloxacillin alongside other agents).

Antibiotics prescribed as part of the first treatment regimen were given for a median (IQR) length of 2 (1–5) days (Table 3). The median (IQR) length of the complete course of antibiotics (first three regimens combined) was 14 (8–28) days. In total, 74% (*n* = 107/145) of patients in this study group received their first antibiotic regimen through the IV route and 67% (*n* = 72/107) of these patients were later switched to oral antibiotics with a median (IQR) time to switch of 4 (3–8) days.

**Table 3.** Antibiotic treatment regimens for patients diagnosed with ABSSSI.

Number of treatment regimens per patient	*n* (%)
1	30 (21)
2	57 (39)
3	31 (21)
⩾4	27 (19)
Length of antibiotic course per regimen (days)	Median (IQR)
First regimen	2 (1–5)
Second regimen	3 (2–7)
Third regimen	6 (2–8)
Total course length for first three regimens	14 (8–28)
Route of administration of first regimen antibiotic	*n* (%)
IV	107 (74)
Oral	28 (19)
Combination of IV and oral	7 (5)
Not known	3 (2)
Time for switch to oral antibiotic (days), *n* (%)	72 (67)
0 ⩽ 5	45 (31)
5 ⩽ 10	13 (9)
>10	12 (8)
Not known	2 (1)

ABSSSI, acute bacterial skin and skin structure; IQR, interquartile range; IV, intravenous.

A post hoc exploratory analysis was carried out to investigate the ‘appropriateness’ of the first-line regimen choice according to a patient’s Eron severity classification, as per criteria published by Marwick and colleagues.^8^ The rationale for ‘appropriateness’ of antibiotics as described by Marwick and colleagues was based on the CREST^18^ guidelines and further expanded to classify any antibiotics which may not fall within the CREST guidelines (Table 4). These criteria were applied to patients who had both Eron and SIRS severity assessment available (*n* = 85). Suitability of antibiotic regimens according to severity classification is described in Table 5. In total, 85% (*n* = 34/40) of patients with an Eron 1 classification were judged to be ‘over-treated’ (i.e. given IV antibiotic), while 15% (*n* = 6/40) patients received an ‘appropriate’ first-line regimen. Out of the 10 patients with Eron class 1 (*n* = 10/34) who also met the criteria for SIRS, 80% (*n* = 8/10) received IV antibiotics initially. For the 43 patients classified as Eron 2, 60% (*n* = 26/43) were judged to have received ‘appropriate’ treatment, 23% (*n* = 10/43) were ‘over-treated’ (dual or triple antibiotic therapy), while 14% (*n* = 6/43) were under-treated (oral therapy or lack of *S. aureus* cover). We also analysed the LOS of inpatients in this subgroup (*n* = 40/85) according to the status of their infection and ‘appropriateness’ of treatment (Supplementary Table 6). Overall, patients with SIRS were more frequently ‘over-treated’ and spent a longer duration of time in hospital.

**Table 4.** Criteria described by Marwick and colleagues for ‘appropriateness’ of antimicrobial treatments for skin and soft tissue infection (SSTI).^8^.

Eron classification	Appropriate antimicrobial therapy
Class 1	Appropriate: oral therapy active against *Streptococcus pyogenes* and *Staphylococcus aureus.* Over-treatment: any IV therapy or oral therapy with unnecessarily broad-spectrum cover. Under-treatment: oral therapy not sufficiently active against *S. pyogenes* and *S. aureus.*
Class 2	Appropriate: IV therapy active against *S. pyogenes* and *S. aureus.* Over-treatment: IV therapy with unnecessarily broad-spectrum cover. Under-treatment: any oral therapy or IV therapy that is not sufficiently active against *S. pyogenes* and *S. aureus*
Class 3	Appropriate: IV therapy active against *S. pyogenes* and *S. aureus.* Over-treatment: IV therapy with unnecessarily broad-spectrum cover. Under-treatment: any oral therapy OR IV therapy that is not sufficiently active against *S. pyogenes* and *S. aureus*.
Class 4	Appropriate: IV therapy active against *S. pyogenes, S. aureus*, Gram-negative bacteria and anaerobic bacteria which included a drug which reduces toxin production by *S. pyogenes* (clindamycin or linezolid^19^) Under-treatment: any oral therapy or IV therapy not sufficiently active against the full range of potential pathogens and their toxins.

IV, intravenous.

**Table 5.** Appropriateness of antimicrobial treatment for patients with different ABSSSI severity classifications.

Eron class^a^	SIRS	*n*	Number of antibiotic regimens, *n* (%)	First-regimen IV antibiotics, *n* (%)	First-regimen Oral antibiotics, *n* (%)	Marwick and colleagues classification of treatment ‘appropriateness’,^b^ *n* (%)
1	SIRS		1: 3 (30%)			Appropriate: 2 (20%) Over-treatment: 8 (80%) Under-treatment: 0 (0%)
		10	2: 2 (20%)	9 (90%)	1 (10%)	
			⩾3: 5 (50%)			
	Non-SIRS		1: 4 (13%)			Appropriate: 4 (13%) Over-treatment: 26 (87%) Under-treatment: 0 (0%)
		30	2:16 (53%)	26 (87%)	4 (13%)	
			⩾3: 10 (33%)			
2	SIRS		1: 1 (7%)			Appropriate: 7 (47%) Over-treatment: 7 (47%) Under-treatment: 1 (7%)
		15	2: 4 (27%)	15 (100%)	0	
			⩾3: 10 (67%)			
	Non-SIRS		1: 5 (18%)			Appropriate: 19 (68%) Over-treatment: 3 (11%) Under-treatment: 5 (18%)
		28^c^	2: 11 (39%)	22 (79%)	5 (18%)	
			⩾3: 12 (43%)			
3	SIRS		1: 0 (0%)			Appropriate: 1 (50%) Over-treatment: 1 (50%) Under-treatment: 0 (0%)
		2	2: 1 (50%)	2 (100%)	0	
			⩾3: 1 (50%)			
	Non-SIRS	0	N/A	N/A	N/A	N/A

ABSSSI, acute bacterial skin and skin structure; SIRS, systemic inflammatory response syndrome; IV, intravenous.

a *n* = 0 for Eron 4.

b First-regimen antibiotics classified according to previous published criteria by Marwick and colleagues.^8^

c One patient could not be assessed for ‘appropriateness’ of treatment due to insufficient data regarding route of administration.

### Healthcare resource utilisation

Management settings for all patients at the time of presentation with ABSSSI and during treatment for ABSSSI are shown in Supplementary Figure 1. A total of 20% (*n* = 14/71) of inpatients were deemed suitable to receive OPAT in the opinion of the attending clinical team. OPAT was available, but not utilised for 13 of the 14 patients. These 14 patients utilised 145 ABSSSI-related inpatient bed days costing an estimated £67,758 (£4840 per patient at £467.30 per day). Treatment in the OPAT setting for these patients would have cost approximately £1383 (£26 per contact, based on mean of 3.8 contacts per patient), representing potential cost savings ranging from £5158 (£368 per patient; assuming 1 inpatient day per patient replaced) to £66,375 (£4741 per patient; assuming all inpatient bed days replaced by OPAT).^17^

At the time of discharge from secondary care responsibility, 43% patients (*n* = 62/145) had a resolved infection, 56% (*n* = 81/145) a resolving infection and 2 patients died in hospital. The median (IQR) ABSSSI-related LOS under secondary care responsibility was 8 (4–15) days (including those treated in non-inpatient settings). The length of hospital stay for patients described according to antibiotic regimens and severity classification is shown in Table 2. Discharge from hospital was delayed for 17% (*n* = 24/143) of patients. Discharge of 54% (*n* = 13/24) of these patients was delayed while waiting for social support.

## Discussion

This prospective observational study of patients with ABSSSI primarily comprised patients who (in the attending clinicians’ opinion) were either unsuitable for initiation or in continuation with beta-lactam antibiotics or were diagnosed with MRSA infections. A wide range of antibiotics (23 different antibiotic regimens were prescribed to patients in this study) and multiple treatment regimens for ABSSSI were used, highlighting the complexity and heterogeneity of ABSSSI management and the absence of a standardised approach to treatment. Majority of patients received their first-regimen antibiotic through the IV route before being switched to oral antibiotics. These issues have been reported in previously published studies on ABSSSI and SSTI treatment and management.^2,8,20^

In this study, we attempted to compare the Eron classification system with the SIRS criteria for assessment of ABSSSI severity. There was little, if any, spontaneous recording of Eron classification in routine clinical practice, and most of the Eron classifications for the patients enrolled in this study were recorded by healthcare professionals when prompted by the study protocol. All clinical components required for the SIRS criteria were available for 83% of the study sample. A significantly greater proportion of patients with SIRS received inpatient therapy. Patients with SIRS also spent a greater length of time in hospital in comparison to patients without SIRS. And, 10 of the 40 patients with Eron class 1 infection had SIRS, suggesting they were misclassified and raising concerns about the ease of use and objectivity of the Eron classification tool.

In this study, we classified treatment ‘appropriateness’ as described by Marwick and colleagues.^8^ Appropriateness of treatment was based on the Eron criteria, but our analysis showed that Eron classifications were not necessarily reliable, for example, in patients who were also diagnosed with SIRS or to patients unable to tolerate or absorb oral agents. While the judgement of appropriate antimicrobial treatment may be subject to assessor bias, these data underscore the need for a common standardised system for ABSSSI severity assessment and appropriate treatment guidelines. Appropriate assessment of ABSSSI severity is imperative for determining optimal antimicrobial treatment strategies.

The median length of first regimen of antibiotics for patients in this cohort was 2 days. This is particularly noteworthy given that several recent studies suggest 72 h as a suitable time-frame for evaluating clinical improvement,^21–23^ and response to antimicrobial treatment within 72 h is also a new Food and Drug Administration (FDA)-recommended endpoint for clinical trials.^14^ It is possible that response to therapy is being assessed too soon, promoting premature changes to therapy and unnecessarily prolonging treatment. The analysis of healthcare resource utilisation in this sample population demonstrated that opportunities for more patients with ABSSSI to receive their antibiotic medication at home or in an outpatient setting exist. Further research will be required to confirm these cost calculations and to also determine the impact of current severity assessment criteria on treatment outcomes in patients with ABSSSI.

### Limitations and generalisability

Data for this observational study were collected from medical records used in routine clinical practice and, as a result, relied on the accuracy and completeness of these records. The fact that the original target sample size of 300 was not achieved may have adversely affected the precision of the outcome variables described in this study. In total, 80% of patients in our study group were diagnosed with cellulitis, but we studied a subset of patients with ABSSSI who were unsuitable for treatment with beta-lactam antibiotics. The predominance of patients with more severe ABSSSI in this study may mean that the findings reported here are not generalisable to all patients with this condition. Several patients who received a beta-lactam antibiotic initially but subsequently switched to non-beta-lactam antibiotics were included in the study cohort. While this may appear prima facie to be in contradiction with the selection criteria, a follow-up of their subsequent treatment and management on non-beta-lactam antibiotics was deemed to be pertinent. The retrospective allocation of SIRS categorisation and the limited sample size in the comparative analysis of clinical outcomes in patients with Eron classification may pose limitations to a wider interpretation of these results. Further studies in larger cohorts of patients with ABSSSI, directly comparing various severity assessments and associated treatment strategies, would be needed to further clarify the clinical impact of different severity assessments.

## Conclusion

This study highlights the current variability in real-world practices for assessment of severity and anti-microbial treatment of ABSSSI where first-line beta-lactam therapy is unsuitable. There is an urgent need for a more robust and standardised approach for the assessment of ABSSSI severity, which is currently subjective and heterogeneous. In particular, where treatments with beta-lactam antibiotics is unsuitable, focused AMS strategies would greatly help in optimising antimicrobial treatment and support the effective use of healthcare resources.

## Supplemental Material

SUPPLEMENTARY_INFORMATION_R_1_OCT_2018 – Supplemental material for Opportunities for antimicrobial stewardship in patients with acute bacterial skin and skin structure infections who are unsuitable for beta-lactam antibiotics: a multicenter prospective observational study Click here for additional data file. Supplemental material, SUPPLEMENTARY_INFORMATION_R_1_OCT_2018 for Opportunities for antimicrobial stewardship in patients with acute bacterial skin and skin structure infections who are unsuitable for beta-lactam antibiotics: a multicenter prospective observational study by Jonathan A.T. Sandoe, Kordo Saeed, Achyut Guleri, Kieran S. Hand, Ryan Dillon, Mike Allen, Amazigom Mayes, Fiona Glen and Armando Gonzalez-Ruiz in Therapeutic Advances in Infectious Disease
