# Supplementary material for: Opportunities for antimicrobial stewardship in patients with acute bacterial skin and skin structure infections who are unsuitable for beta-lactam antibiotics: a multicenter prospective observational study
Source: Ther Adv Infect Dis. 2019 Feb 4;6:2049936118823655. doi: 10.1177/2049936118823655 (PMC6365991; doi:10.1177/2049936118823655)
Supplement: SUPPLEMENTARY_INFORMATION_R_1_OCT_2018 – Supplemental material for Opportunities for antimicrobial stewardship in patients with acute bacterial skin and skin structure infections who are unsuitable for beta-lactam antibiotics: a multicenter prospective observational study [file SUPPLEMENTARY_INFORMATION_R_1_OCT_2018.docx]

# SUPPLEMENTARY INFORMATION

**Table 1** **Eron classification of skin and soft tissue infection (SSTI) according to the severity of local and systemic signs, and associated management[4]**

| **Category** | **Clinical features** |
| --- | --- |
| Class 1 | SSTI with no signs or symptoms of systemic toxicity or comorbidities |
| Class 2 | Either systemically unwell or systemically well but with comorbidity that may complicate or delay resolution |
| Class 3 | Toxic and unwell (fever, tachycardia, tachypnoea and/or hypotension) |
| Class 4 | Sepsis syndrome and life-threatening infection (e.g. necrotising fasciitis) |

**Table 2 Criteria for Systemic Inflammatory Response Syndrome[7]**

| **Two or more of:** | Temperature >38°C or <36°C |
| --- | --- |
|  | Heart rate >90/min |
|  | Respiratory rate >20/min or PaCO_2_ <32mmHg (4.3 kPa) |
|  | White blood cell count >12 000/mm^3^ or <4000/mm^3^ or >10% immature bands |

**Table 3 Antibiotics prescribed as first regimen treatment for ABSSSI**

| **First regimen antibiotics** | **Narrow spectrum**: active against *Staphylococci* and *Streptococci*.  **Broad spectrum**: active against *Staphylococci and Streptococci*, Gram-negatives and anaerobes including toxin inhibitor |
| --- | --- |
| Teicoplanin | **Staphylococci and Streptococci* |
| Clindamycin | *Staphylococci and Streptococci* and anti-toxin |
| Flucloxacillin | *Staphylococci and Streptococci* |
| Vancomycin | **Staphylococci and Streptococci* |
| Clarithromycin | *Staphylococci and Streptococci* |
| Gentamicin | Gram-negative agent (part of broad-spectrum regimen) |
| Metronidazole | Anti-anaerobe agent (part of broad-spectrum regimen) |
| Linezolid | **Staphylococci and Streptococci* |
| Benzylpenicillin | *Streptococci* only |
| Ceftriaxone | *Staphylococci, Streptococci* and Gram-negative cover (broad-spectrum) |
| Cefuroxime | Staphs, Streps and Gram-negative cover (broad-spectrum) |
| Daptomycin | **Staphylococci and Streptococci* |
| Amoxicillin | *Streptococci* only |
| Co-trimoxazole | * *Staphylococci, Streptococci* and Gram-negative cover (broad-spectrum) |
| Moxifloxacin | *Staphylococci, Streptococci* and Gram-negative cover (broad-spectrum) |
| Rifampicin | * *Staphylococci, Streptococci* and anti-toxin |
| Amikacin | Gram-negative agent (part of broad-spectrum regimen) |
| Erythromycin | *Staphylococci and Streptococci* |
| Ciprofloxacin | Gram-negative agent (part of broad-spectrum regimen) |
| Piperacillin/tazobactam | *Staphylococci and Streptococci* and Gram-negative cover (broad-spectrum) |
| Doxycycline | **Staphylococci and Streptococci* and Gram-negative cover (broad-spectrum) |
| Cefotaxime | *Staphylococci, Streptococci* and Gram-negative cover (broad-spectrum) |
| Co-amoxiclav | *Staphylococci, Streptococci* and Gram-negative cover (broad-spectrum) |

** includes MRSA cover; beta-lactam antibiotics are shown in grey rows*

**Table 4 Reasons for change in antibiotics regimen**

| **Reasons of choice of 1st therapy*** | **Number of patients** | **% (n=145)** |
| --- | --- | --- |
| Clinician choice | 45 | 31% |
| Allergy | 39 | 27% |
| Severity of infection | 29 | 20% |
| Previous treatment failure | 18 | 12% |
| IV no longer a requirement | 5 | 3% |
| Result of C&S | 3 | 2% |
| Unsuitable for oral therapy | 1 | 1% |
| Hospital/treatment policies and guidelines | 14 | 10% |
| Other | 8 | 6% |
| **Reasons based on clinician’s judgement, not mutually exclusive* |  |  |

**Table 5 Distribution of patients receiving various first regimen antibiotics**

| **Antibiotics used as part of first regimen** | **Number of patients*** | **% (n=145)** |
| --- | --- | --- |
| Teicoplanin | 47 | 32% |
| Clindamycin | 47 | 32% |
| Flucloxacillin | 26 | 18% |
| Vancomycin | 15 | 10% |
| Clarithromycin | 10 | 7% |
| Gentamicin | 8 | 6% |
| Metronidazole | 6 | 4% |
| Benzylpenicillin | 4 | 3% |
| Ceftriaxone | 4 | 3% |
| Cefuroxime | 4 | 3% |
| Other beta-lactam | 3 | 3% |
| Other non-beta-lactam | 18 | 3% |

**Numbers are not mutually exclusive*

**Table 6 LOS of inpatients with SIRS classification according to ‘appropriateness’ of treatment**

| **Median (IQR) LOS*** | **Appropriate** | **Over-treated** |
| --- | --- | --- |
| **SIRS** | 6.0 (4.0-13.0) n=7 | 19.5 (14.8-38.5) n=12 |
| Resolved infection | 13.0 (9.5 - 22.3) n=4 | 19.5 (16.0 - 33.5) n=6 |
| Resolving | 4.0 (3.0-5.0) n=2 | 20.0 (7.8-35.3) n=6 |
| **Non SIRS** | 12.0 (10.8-14.8) n=8 | 11.0 (4.0-19.0) n=10 |
| Resolved infection | 12.5 (9.5-16.0) n=4 | 12.0 (7.5-20.5) n=8 |
| Resolving | 12.0 (11.0-12.0 ) n=3 | 4.0 (3.0-5.0) n=2 |
| **Infection not resolved for 2 patients receiving 'appropriate' treatment (1 SIRS, 1 non SIRS)* | | |

**Figure 1 Management settings for patients diagnosed with ABSSSI**


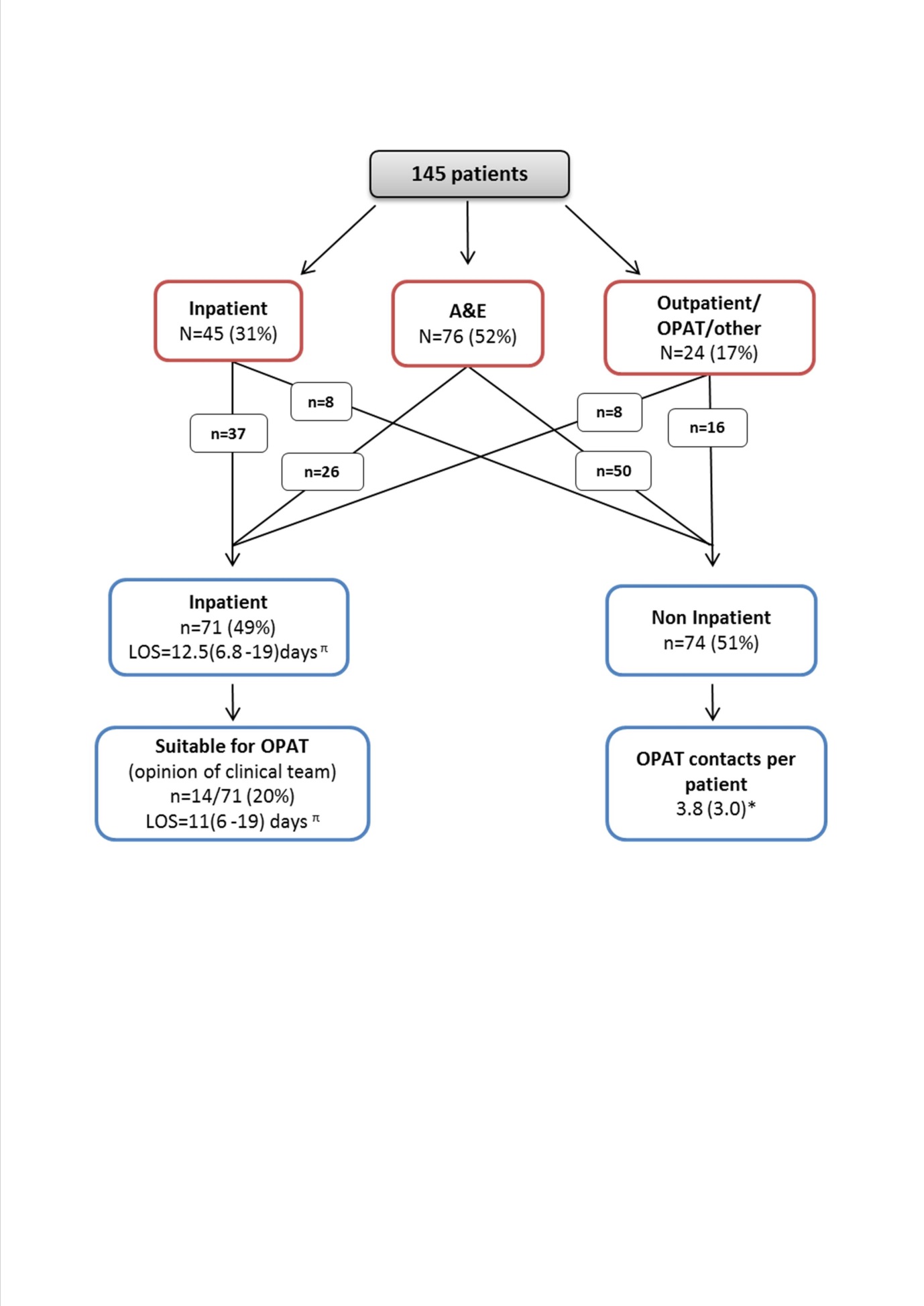


*^π^Result expressed as Median (interquartile range),*Results shown as Mean (SD). LOS is ABSSSI-related length of stay i.e. number of days until patient was deemed fit for discharge from under secondary care responsibility by the clinical team. Red boxes show patient management settings at the time of ABSSSI presentation and blue boxes represent management settings during treatment of ABSSSI.*
